# Risk factors for the development of tuberculosis among the pediatric population: a systematic review and meta-analysis

**DOI:** 10.1007/s00431-023-04988-0

**Published:** 2023-05-02

**Authors:** Nayana Siddalingaiah, Kiran Chawla, Sharath Burugina Nagaraja, Druti Hazra

**Affiliations:** 1 Department of Microbiology, Kasturba Medical College Manipal, Manipal Academy of Higher Education (MAHE), Manipal, 576104 India; 2 Department of Community Medicine, Employees State Insurance Corporation Medical College and Post Graduate Institute of Medical Sciences and Research, Bengaluru, 560010 India

**Keywords:** Pediatric, Tuberculosis, Risk factors, High-burden countries

## Abstract

Pediatric tuberculosis is a major cause of mortality and morbidity in children due to high transmission, poor diagnostic tools, and various respiratory diseases mimicking TB. Identifying risk factors will provide evidence for clinicians to strongly relate their diagnosis to the associated pathology. Studies were retrieved from PubMed, Embase, and Google Scholar, systematically reviewed, and meta-analyzed for various risk factors and their association with pediatric TB. Meta-analysis depicted that four out of eleven risk factors were significant—contact with known TB cases (OR 6.42 [3.85,10.71]), exposure to smoke (OR 2.61 [1.24, 5.51]), overcrowding in the houses (OR 2.29 [1.04, 5.03]), and, poor household conditions (OR 2.65 [1.38, 5.09]). Although significant odds ratio estimates were obtained, we observed heterogeneity in the studies included.

*    Conclusion*: The study findings demand the constant screening of risk factors such as contact with known TB cases, exposure to smoke, overcrowding, and, poor household conditions for the development of pediatric TB.

**Table:** 

**What is Known:** *• Knowledge of the risk factors of a disease is of utmost importance in the planning and institution of its control measures. Well-established risk factors in the occurrence of TB in the pediatric group are HIV positivity, older age and close contact with a known case of TB*.	
**What is New:** *• In addition to what is already known; this review and meta-analysis has identified exposure to indoor smoking, overcrowding and poor household conditions as important risk factors for developing pediatric TB*. *• Implications of the study: The findings highlight that in addition to routine contact screening for the pediatric group, the children living in poor household conditions and getting exposed to passive indoor smoking demand more attention to prevent the development of pediatric TB.*

**Supplementary Information:**

The online version contains supplementary material available at 10.1007/s00431-023-04988-0.

## Introduction

Globally, the prevention and management of childhood tuberculosis (TB) have posed to be a major challenge to clinicians, academicians, and program managers for decades. Programmatically, TB in children has failed to gain enough momentum despite its high mortality and morbidity because the transmission rate from children is low due to the paucibacillary state [1]. The lack of newer TB diagnostic tools that are simple and feasible to implement in the field adds up to the existing problem. The incidence of pediatric TB is the direct measure and a proxy indicator for the transmission of TB in the community [1, 2].

The Global TB Report 2021 states that 8.75% of the total treated cases of TB during 2018–2022 were children (3.5 out of 40 million) signifying that the transmission rate is relatively high in the community [3]. Hence, the risk factors leading to the development of TB disease have gained focus over the years. Children bear severe forms of TB in comparison to adults [4, 5]. Since no tests are available to measure the progression of the disease, the associated risk factors can be advantageous to speculate the disease.

Contact with known adult TB cases is known as an important risk factor in the case of both children and adults, whereas other risk factors are less explored in the pediatric population [6, 7]. The Global TB Report 2020 suggests that 86% of newly detected pediatric cases occurred in 30 high-burden countries [8, 9]. Thus, this study is focused on comprising research data published in these high-burden countries listed by the World Health Organization (WHO).

To address the knowledge gaps, data were pooled from studies published from January 1990 to June 2022 and systematically reviewed to determine the association of various risk factors with the development of TB among the pediatric population.

## Methods

This systematic review protocol was registered at the International Prospective Register of Systematic Reviews (PROSPERO) (identifier: CRD42022315722), and the study was conducted and reported as per the Meta-analysis of Observational Studies in Epidemiology (MOOSE checklist) [10]. The MOOSE checklist is provided as supplement copy 1.

### Search strategy and selection criteria

We searched electronic databases like MEDLINE (through PubMed), Embase, and Google Scholar for the published research articles, limited to the English language from January 1990 to June 2022, reporting the risk factors for tuberculosis in children less than 19 years old.

The search strategy was developed with combinations of keywords like pediatric, tuberculosis, risk factors, and the list of high TB burden countries along with their synonyms, Boolean operators, and truncations as per the databases.

In the PubMed, we used the search term “pediatric*”[All Fields] OR “pediatrics”[MeSH Terms] OR “paediatric*”[All Fields] OR “child*”[All Fields] OR “child”[MeSH Terms] OR “adolescent”[MeSH Terms] OR “adolescen*”[All Fields] OR “infant*”[All Fields] OR “infant”[MeSH Terms] OR “infant, newborn”[MeSH Terms] OR “newborn*”[All Fields] “tuberculosis”[All Fields] OR “tuberculosis”[MeSH Terms] OR “tuberculosis”[All Fields] OR “tuberculosis”[MeSH Terms] OR “TB”[All Fields] OR “Latent TB”[All Fields] OR “latent tuberculosis”[MeSH Terms] OR “Koch’s Disease”[All Fields] OR “mycobacterium infections”[MeSH Terms] OR “*Mycobacterium tuberculosis*”[MeSH Terms] OR “*Mycobacterium tuberculosis*”[All Fields] “risk factor*”[All Fields] OR “risk factors”[MeSH Terms] OR “socioeconomic factor*”[All Fields] OR “social determinants of health”[MeSH Terms] OR “social determinant*”[All Fields] OR “risk determinant*”[All Fields] OR “risk predictor*”[All Fields] OR “epidemiologic factor*”[All Fields] OR “epidemiologic factors”[MeSH Terms] OR “risk assessment*”[All Fields].

A similar search strategy was used for Embase and Google Scholar, which is provided in supplement copy 2.

### Screening and selection

The research articles were imported into the Rayyan website (https://www.rayyan.ai/) and duplicates were removed. Two investigators independently screened the title and abstract of the articles based on the following inclusion and exclusion criteria.

#### Inclusion criteria

We included case–control, cohort, and cross-sectional studies with the pediatric population less than 19 years old and reported risk factor analysis for TB.

#### Exclusion criteria

Reviews and conference abstracts were excluded. Study outcomes focusing on the adult population and pediatric cases with previously known contact with TB were excluded.

Subsequently, the authors reviewed full-text versions of selected records. The reasons for article exclusion were recorded, and potential disagreements were specified to be resolved by the involvement of a third investigator (BNS).

### Data extraction

The data extracted from the included articles were entered into a standardized excel sheet. The data sheet included variables such as author, year of publication, study design, the country where the study was conducted, study group, sample size, and significant risk factors.

### Quality assessment

Quality assessment was done by the Newcastle–Ottawa scale to check the methodological quality of the selected articles [11]. Studies scoring ≥ 7 points were considered high quality, 5–6 were medium quality, and ≤ 4 was low quality.

### Data analysis

Meta-analysis of the studies was depicted as forest plots to estimate the overall odds ratio (OR). Heterogeneity among studies was evaluated using the Galbraith plot and *I*^2^ statistic categorized as follows: < 30% not important, 30–50% moderate, 50–75% substantial, and 75–100% considerable. If significant heterogeneity was identified among studies, further examination of the individual studies was conducted, and random effects models (Dersimonian-Laird method) were used to obtain the summary odds ratio estimates. Otherwise, fixed effects models were used (Mantel–Haenszel method). Publication bias was assessed by the Egger regression asymmetry test and funnel plots. The meta-analytic software program used was Stata 17.0 BE-Basic (S. No. 301706309069).

## Results

### Search results

**Fig. 1:**
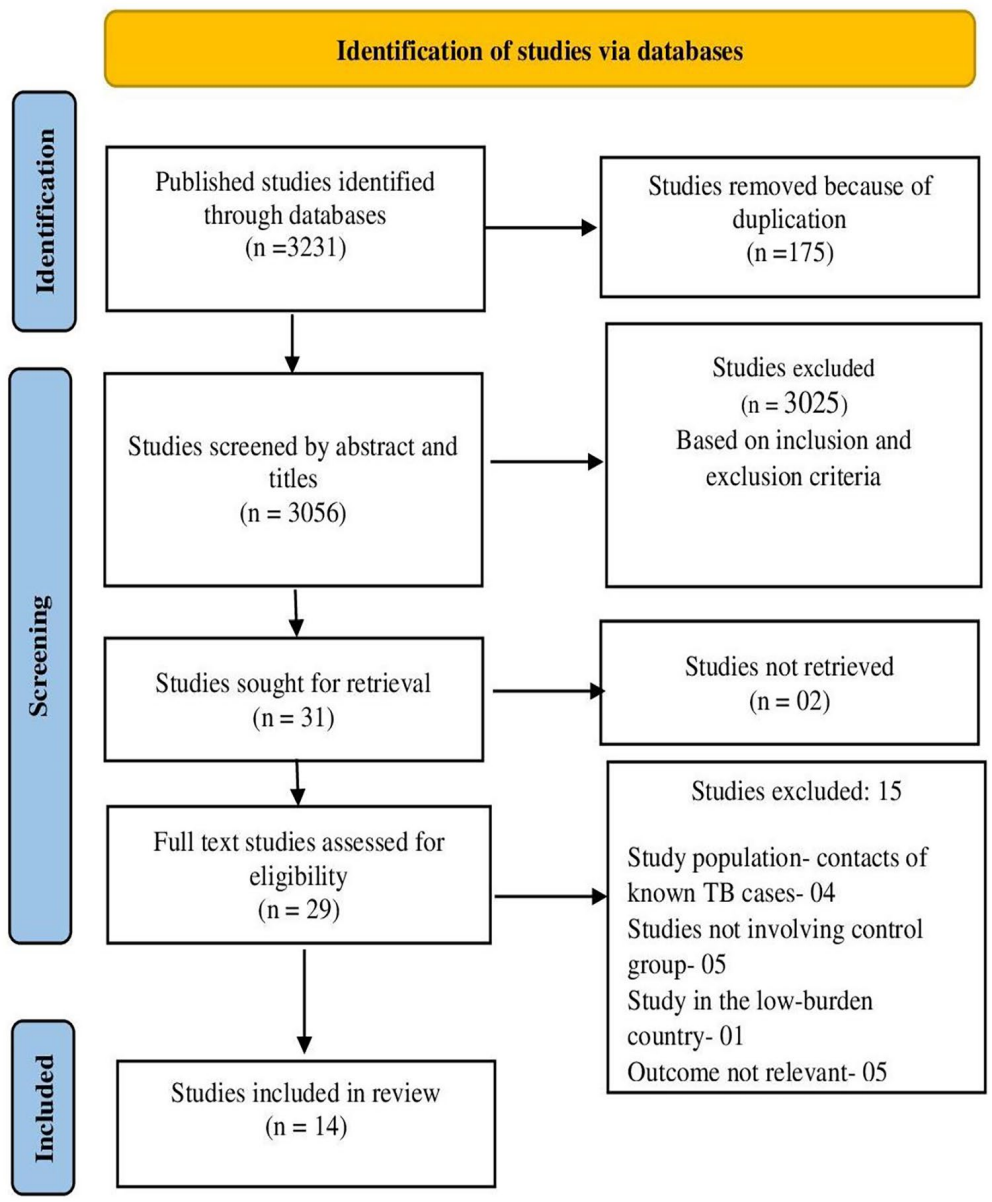
PRISMA flow diagram showing literature search and screening process

Based on the keyword search in the database of PubMed, Embase, and Google Scholar, we obtained 3231 articles. Of these, 3056 articles were screened for titles and abstracts after excluding 175 duplicates. The studies were listed based on our study inclusion and exclusion criteria; 31 studies were subjected to full-text screening after which 14 eligible studies were included for analysis. Figure 1 illustrates the PRISMA flow chart (https://prisma-statement.org/prismastatement/flowdiagram.aspx) of studies retrieved and the selection process through various stages.

### Characteristics of the studies

Characteristics of included studies are shown in Table 1. All the included studies in this review belong to the eight high TB burden countries: South Africa, Nigeria, Uganda, Brazil, China, the Philippines, Bangladesh, and India. Among these fourteen articles, seven were case–control studies, along with five cross-sectional and two cohort studies. The study population ranged from 0 to 19 years old with the most common age group between 10 and 18 years, and the detection of latent TB infection or TB disease was the aim of most of the articles. Either questionnaire or interview was used as a data collection tool for the studies included. The significant risk factors in the individual study are tabulated.

**Table 1:** General characteristics of the studies in the review

**References**	**Setting**	**Design**	**Diagnostic test used**	**Study group**	**Risk factors**	**Quality assessment**
Mzembe et al., 2021 [12]	South Africa	Cross-sectional study	QFT-Plus	Adolescents (aged 10–19 years)	1. Older age 2. Household TB contact 3. Increasing community-level HIV prevalence	-
Gatchalian et al., 2020 [13]	Bohol, Philippines	Community-based prevalence study	TST and standardized TB disease screening	Children are grouped into age groups below 5 years and ≥ 5 years	1. Being 5 years or older 2. Having a known TB contact 3. Having a known TB contact who was either the mother or another primary caregiver 4. Living in a high TB burden municipality	-
Jafta et al., 2019 [14]	South Africa	Case–control study	Cases were diagnosed as per the national program guidelines^a^ (GeneXpert MTB/RIF or clinical signs and symptoms)	Children aged 0–14 years	1. Presence of dampness in the household	Selection: **** Comparability: * Outcome: ** High quality
Bunyasi et al., 2019 [15]	South Africa	Cohort study	QFT-Plus	Adolescents (aged 12–19 years)	1. Older age 2. Low socio-economic status	Selection: **** Comparability: ** Outcome: *** High quality
Attah et al., 2018 [5]	Nigeria	Cross-sectional descriptive study	Chest X-ray, microscopy for acid-fast bacilli (sputum or gastric aspirate), and mycobacterium culture^a^	Children between the age of 18 months and 15 years	1. Absence of cross ventilation 2. Contact with the adult source case 3. Overcrowding	-
Mumpe-Mwanja et al., 2015 [16]	Eastern Uganda	Prospective cohort study	TST	Adolescents aged 12–18 years	1. BCG scar 2. male gender 3. Older age 4. Being out of school 5. Known history of household TB contact in the last 2 years	Selection: *** Comparability: * Outcome: * Medium quality
Jubulis et al., 2014 [17]	Pune, India	Case–control study	Cases were diagnosed as per the national program guidelines^a^ (microscopy for acid-fast bacilli and culture and chest radiography)	Children aged ≤ 5 years	1. Household TB 2. Household food insecurity 3. Indoor air pollution exposure	Selection: *** Comparability: ** Outcome: ** High quality
Stevens et al., 2014 [18]	Recife, Brazil	Case–control study	Cases were diagnosed as per the national program guidelines^a^ (clinical and laboratory tests, and chest radiography)	Children aged 7 to 19	1. Sleeping in the same house as a case of tuberculosis 2. Living in a house with no piped water (probably as a proxy for bad living conditions) 3. Illiteracy 4. Male sex	Selection: **** Comparability: * Outcome: ** High quality
Hu et al., 2013 [19]	China	Cross-sectional study	T-SPOT.TB	Children and adolescents aged between 10 and 18 years	1. No BCG vaccination 2. History of TB exposure	-
Karim et al., 2012 [20]	Bangladesh	Case–control study	Sputum microcopy positive for acid-fast bacilli	Children below 18 years	1. Older age 2. Household contact Bad living conditions	Selection: *** Comparability: ** Outcome: ** High quality
Patra et al., 2012 [21]	India	Case–control study	Cases were diagnosed as per the national program guidelines^a^	Children aged 0 to 14	1. Education of the mother 2. A family member having tuberculosis in the last 2 years and residing in the same house 3. A passive smoker	Selection: *** Comparability: * Outcome: ** Medium quality
Karim et al., 2012 [22]	Bangladesh	Case–control study	Sputum micrsocopy positive for acid-fast bacilli	Children (< 18 years old)	1. Child more than 14 years of age 2. Respondents’ education—pre-primary 3. Mother’s education—illiterate 4. Father’s occupation—daily laborer 5. More than two persons per room 6. Position of kitchen Contact with TB case	Selection: *** Comparability: * Outcome: ** Medium quality
Mahomed et al., 2011 [23]	South Africa	Prevalence study	TST and QFT-Plus	Adolescents aged 12–18 years	1. Black/mixed race racial groups 2. Male sex 3. Older age 4. Household TB contact 5. Low income 6. Low education level	-
Ramachandra et al., 2011 [24]	South India	Case–control study	Cases were diagnosed as per the national program guidelines^a^	Children of age group 0–14 years	1. LBW 2. Malnutrition 3. Passive smoking 4. Firewood smoke	Selection: **** Comparability: * Outcome: ** High quality

^a^TB disease screening and evaluation are aligned with the respective country’s national guidelines and the study period of the individual research work

### Quality assessment

Quality assessment of the included study methodology was carried out for both cohort and case–control study designs. The quality of cross-sectional studies could not be assessed due to the non-availability of the standardized tool. Criteria for assessing the quality of studies are included in supplement copy 2.

#### Cohort studies

Of the two cohort studies assessed for quality, one each was of high and medium quality.

#### Case–control studies

Out of the seven studies assessed for quality, 71 percent of studies (05 nos.) were high quality, and two studies are of medium quality.

### Risk factors

Among the 14 studies included, the significant risk factors identified were (Table 2) TB contacts (12/14), living conditions (11/14), child health and well-being (07/14), older age (07/14), socio-demographic factors (05/14), socio-economic status (04/14), education (03/14), and food insecurity (02/14). Contact with a known TB case is the most common risk factor among the studies, and food insecurity is the least common risk factor. Contact with TB cases, poor living conditions, child health, and older age have occurred in 50% or more studies whereas socio-demographic factors, socio-economic status, educational status, and food insecurity have occurred in less than 50% of the studies.

**Table 2:** Proportion of risk factors detected

**Risk factor**		**Number of studies (*n*** **= 14)**	**Percentage (%)**
**TB contact**	Relationship with TB case	2	**85.7**
	Household contact	7	
	Exposure to a known adult case	3	
	**Total**	**12**	
**Living conditions**	Overcrowding	3	**78.6**
	In-house environmental factors	6	
	Passive smoking	2	
	**Total**	**11**	
**Child health and well-being**	BCG vaccination	2	**50**
	Literacy	2	
	Chronic allergy-related conditions	1	
	Low birth weight	1	
	Malnutrition	1	
	**Total**	**7**	
**Older age**	**Total**	**7**	**50**
**Socio-demographic factors**	Living in a high-burden TB community	1	**35.7**
	Community HIV prevalence (%)	1	
	Gender	2	
	Racial group	1	
	**Total**	**5**	
**Socio-economic status**	**Total**	**4**	**28.6**
**Education**	Maternal education	2	**21.4**
	Child’s education	1	
	**Total**	**3**	
**Food insecurity**	**Total**	**2**	**14.2**

### Interpretation of study findings

#### Case-control studies (Table 3)

Risk factors that are common in at least two of the studies were selected to calculate the pooled odds ratio. The following factors showed significance when pooled odds ratio at 95% confidence interval was calculated—household contact 6.199 (4.836–7.946), child’s education 0.5293 (0.4018–0.6974), father’s education 0.7607 (0.6158–0.9396), father’s occupation or income 1.349 (1.08–1.684), HIV positivity 22.81 (8.01–64.97), passive smoking 2.352 (1.694–3.265), exposure to indoor gases 2.062 (1.566–2.715), household condition 2.51 (1.966–3.204), less number of rooms 2.755 (1.961–3.87), overcrowding 2.414 (1.898–3.07), no adequate ventilation 2.366 (1.349–4.149), and kitchen position 2.153 (1.611–2.876).

**Table 3:** Pooled odds ratio of the risk factors for case-control studies

**Sl. no.**	**Risk factors**	**Number of studies (*n*= 07)**	**Case**		**Control**		**Pooled odds ratio with 95% CI**
			**Exposure**	**Non-exposure**	**Exposure**	**Non-exposure**	
**1**	**Older age**	07	571	196	929	263	0.8247 (0.667–1.02)
**2**	**Gender**	06	289	278	480	512	1.109 (0.9021, 1.363)
**3**	**Household TB contact**	07	286	445	107	1032	6.199 (4.836–7.946)^*^
**4**	**Contact with TB cases other than household**	03	114	201	197	457	1.316 (0.9903–1.748)
**5**	**Child’s education**	04	291	126	637	146	0.5293 (0.4018–0.6974)^*^
**6**	**Mother’s education**	06	284	312	310	400	1.175 (0.9438–1.462)
**7**	**Father’s education**	05	217	383	397	533	0.7607 (0.6158–0.9396)^*^
**8**	**Mother’s occupation**	03	195	36	230	40	0.942 (0.5777–1.536)
**9**	**Father’s occupation**	06	249	327	271	480	1.349 (1.08–1.684)^*^
**10**	**Child’s HIV status**	02	53	97	4	167	22.81 (8.01–64.97)^*^
**11**	**Child’s weight or BMI**	02	45	56	83	116	1.123 (0.6928–1.82)
**12**	**Passive smoking**	03	122	166	95	304	2.352 (1.694–3.265)^*^
**13**	**Exposure to indoor air pollution**	04	176	229	142	381	2.062 (1.566–2.715)^*^
**14**	**Household condition**	06	184	377	161	828	2.51 (1.966–3.204)^*^
**15**	**Fewer number of rooms**	03	219	78	159	156	2.755 (1.961–3.87)^*^
**16**	**Overcrowding**	05	355	183	266	331	2.414 (1.898–3.07)^*^
**17**	**No adequate ventilation**	02	36	112	25	184	2.366 (1.349–4.149)^*^
**18**	**BCG vaccination**	03	16	285	26	374	0.8076 (0.4252, 1.534)
**19**	**Kitchen position**	03	203	185	131	257	2.153 (1.611–2.876)^*^

^*^Significant OR estimates

#### Cohort studies

Out of the 14 studies selected for review, 2 studies were cohort study designs. Bunyasi et al. [15] reported 48.5% of LTBI in comparison to the prevalence a decade ago which was 43.8%. Mumpe-Mwanja et al. [16] reported 16.1% LTBI. The average LTBI prevalence among these studies was 36.1%.

#### Cross-sectional studies

- Mzembe et al. [12] reported a 23% MTB infection prevalence using a QFT assay in rural South Africa.
- Gatchalian et al. [13] reported 6.5% of TST positivity and 0.3% had active clinical TB disease in the Philippines.
- Attah et al. [5] reported 32% of definitive TB cases in Nigeria.
- Hu et al. [19] reported that 4.7% had positive T-SPOT in Shanghai, China.
- Mahomed et al. [23] reported that 55.2% had positive TST tests and 50.9% had positive QFT in South Africa.

### Meta-analysis

**Fig. 2:**
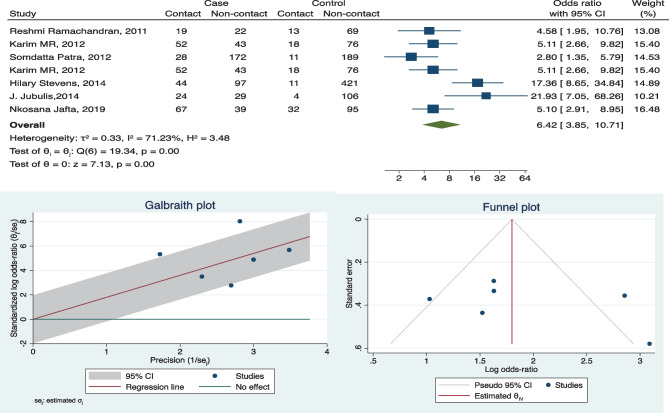
Forest plot, Galbraith plot, and funnel plot for TB contact as a risk factor

**Fig. 3:**
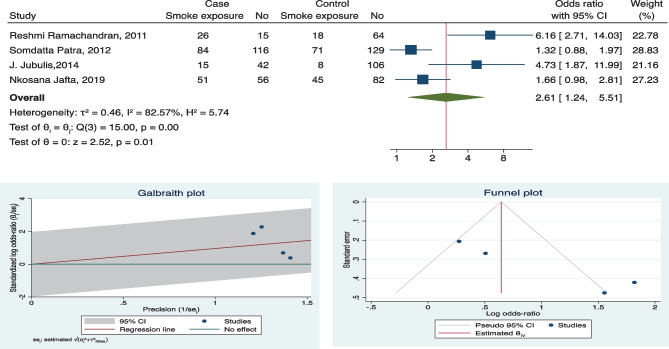
Forest plot, Galbraith plot, and funnel plot for exposure to smoke as a risk factor

**Fig. 4:**
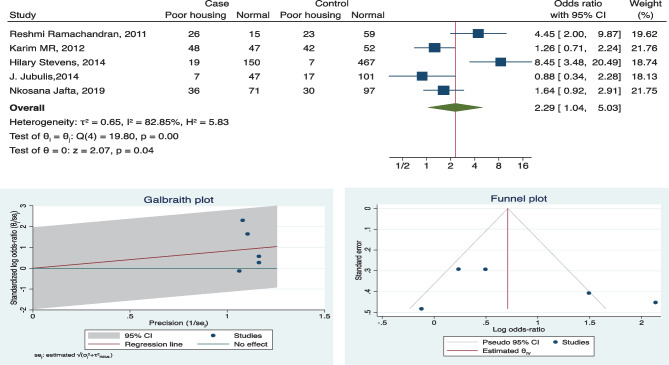
Forest plot, Galbraith plot, and funnel plot for housing condition as a risk factor

**Fig. 5:**
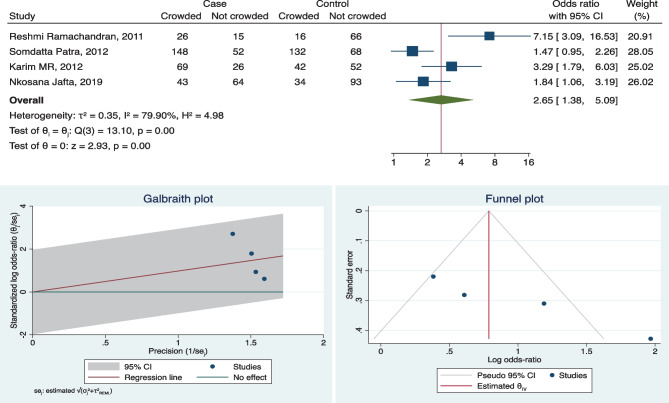
Forest plot, Galbraith plot, and funnel plot for crowded house as a risk factor

We considered eleven factors for the meta-analysis; out of these four risk factors are household TB contact OR 6.42 (3.85, 10.71), exposure to smoke OR 2.61(1.24, 5.51), poor housing condition OR 2.29 (1.04, 5.03), and crowding OR 2.65 (1.38, 5.09) which showed overall significance for developing TB (refer to forest plot in Figs. 2, 3, 4 and 5 and supplement copy 2 for additional forest plots).

### Heterogeneity

Galbraith plots were used to assess the heterogeneity of the study, which showed significant heterogeneity. Most of the included studies in this review showed inconsistency; ten out of the eleven factors considered for meta-analysis show heterogeneity above 50%. Since moderate to considerable values of heterogeneity are observed, the study finding will not be conclusive (refer to Galbraith plots in Figs. 2, 3, 4, and 5 and supplement copy 2 for additional Galbraith plots).

### Publication bias

Funnel plots were used to assess publication bias, despite four factors being overall significant but do not appear to contain symmetric data. Few studies fall out of the confidence interval range as well (refer to funnel plots in Figs. 2, 3, 4, and 5 and supplement copy 2 for additional funnel plots).

## Discussion

Our study focused on the risk factors associated with TB among the pediatric population. Fourteen studies matched our inclusion criteria. Seven studies were included to analyze the pooled odds ratio.

The studies were categorized based on the year of publication, and the characteristics of each study were tabulated. The significant risk factors of all the studies were grouped under a broad category, and the frequency of each risk factor that occurred in all the studies was recorded. The quality of the studies was checked by the Newcastle–Ottawa scale where most of the studies included in our study are good quality. The odds ratio was calculated for the risk factors of the case–control studies, where thirteen out of the nineteen factors considered for the analysis were significant.

The risk factors that appeared to be significant in the calculated pooled odds ratio are described in detail below:

1. Gender
  In our review, the odds of developing TB in males compared to females were non-significant. Two included studies (Mumpe-Mwanja et al. and Stevens et al.) reported males as the risk factor, whereas the finding of our study is contradicting. Also, very few studies had evaluated gender as a risk factor in childhood TB [25, 26]. Gender can be considered a risk factor only after the child attains puberty since sexual hormones play an important role in immunological dimorphism [27]. It is well established in adults that males have a higher risk of TB as they encounter the outside world more often as the breadwinner of the family [25].
2. Household contact
  The odds of household contact developing TB, when compared to no contact or external contact, were found to be five times higher in our review. Twelve out of the fourteen studies had adult case exposure as the risk factor, but the exposure varied from household and non-household. Hence, we analyzed the known TB case exposure in two categories household contact and non-household contact. A study conducted by Jaganath et al. in Uganda showed a 10% TB prevalence among child contacts [28]. Low detection in contacts might be due to low screening. In a similar study conducted by Laghari et al. in India, only 9.3% of contacts were screened [29]. Our earlier research findings have also highlighted the need for constant screening for early diagnosis of pediatric TB cases [30].
3. Child’s education
  According to our findings, the odds of school-going children developing TB are insignificant compared to the children not attending school. Children going to school and/or with higher educational qualifications had lower chances of acquiring the disease since they might have a better knowledge of disease transmission and causes of the diseases like TB. Two studies (two articles published by Karim et al.) reported that the incidence of TB in children who attended school till higher classes are relatively low compared to children who skipped school or discontinued during primary education. A study conducted in Malawi reported that 90% of the children know about the TB disease and its transmission while a lower proportion of children were aware of the symptoms [31]. Children should be encouraged to attend school and continue their higher education which will increase disease awareness.
4. Father’s occupation/income
  The odds of children with low-income/lower socio-economic households have a 0.3 times higher risk of developing TB compared to the children with stable-income households, as per our review. As described, TB is a disease in poor people, and the occurrence of the disease is directly impacted by the socio-economic status of the family, which rationalized the high burden of TB in low-middle-income countries. Better socio-economic status indirectly caters to the nutritional and healthcare requirements of the child. Consumption of nutritious food at a young age will help in the overall development of the child. Providing protein-rich food through government programs can solve the problem of malnutrition.
5. Passive smoking and exposure to indoor air pollution
  We observed that the odds of children exposed to tobacco smoke and indoor air pollution had two times higher risk of developing TB compared to the children not being exposed. Previous studies have concluded that both passive exposure and active exposure to tobacco smoke are associated with TB disease [32]. Smoking is prevalent in most developing countries; hence, exposure to tobacco smoke can be of great concern as smoking leads to the downregulation of macrophage TNF-α in the lungs [32, 33]. A systematic review published by Patra et al. reported that more than a threefold increased risk of secondhand smoke (SHS) is associated with active TB in children [33]. Toxic particulate matter produced from the burning of biomass induces inflammation in the lungs causing respiratory tract-related infections and asthma. Parents should be counselled about the harmful effects of smoke on their child’s health. Those willing to quit can be sent to rehabilitation centers. In the case where quitting is not an option, smoking inside houses or around children should be avoided. Cooking outside the home if no separate kitchen or use of LPG will reduce the risk of exposure to harmful gases.
6. HIV status
  As per our review, the odds of children living with HIV have a twenty-one times higher chance of developing TB when compared to children with no history of HIV. Dodd et al. concluded that HIV is a potent risk factor for childhood TB, and early diagnosis of HIV with anti-retroviral therapy (ART) initiation could reduce the risk of developing TB [34].
7. Household condition
  The odds of children living in poor household conditions had two times higher risk of developing TB when compared to a normal household as per our study. A cross-sectional study conducted in Thailand to determine the association between environmental factors and TB infection showed that children living in crowded households were five times more likely to have the infection [35]. It is better to suggest the patient be isolated in a well-ventilated room; hence, exposure to other family members will be reduced.

## Conclusion

Contact with known TB cases, exposure to smoke, overcrowding in the houses, and poor household conditions are the significant risk factors for pediatric TB. The public health implications of these findings suggest we keep a constant watch for these factors and focus our intervention accordingly, to reach the goal of TB elimination by 2030.

## Supplementary Information

Below is the link to the electronic supplementary material.

Supplementary file1 (PDF 256 KB)

Supplementary file2 (DOCX 1117 KB)

## Data Availability

All data supporting the findings of this study are available within the paper and its supplementary Information.
