# Supplementary material for: Risk factors for the development of tuberculosis among the pediatric population: a systematic review and meta-analysis
Source: Eur J Pediatr. 2023 May 2;182(7):3007–19. doi: 10.1007/s00431-023-04988-0 (PMC10354179; doi:10.1007/s00431-023-04988-0)
Supplement: Supplementary file 2 — Supplementary file2 (DOCX 1117 KB) [file 431_2023_4988_MOESM2_ESM.docx]

**Complete search strategies for electronic databases**

**PubMed/MEDLINE (NCBI)**

### Records obtained on June 05^th^ 2022- 621

Filters applied- Case Reports, Classical Article, Clinical Study, Comparative Study, Evaluation Study, Multicenter Study, Observational Study, Randomized Controlled Trial, Humans, English, Child: birth-18 years, Newborn: birth-1 month, Infant: birth-23 months, Infant: 1-23 months, Preschool Child: 2-5 years, Child: 6-12 years, Adolescent: 13-18 years, from 1990 - 2022

"pediatric*"[All Fields] OR "pediatrics"[MeSH Terms] OR "paediatric*"[All Fields] OR "child*"[All Fields] OR "child"[MeSH Terms] OR "adolescent"[MeSH Terms] OR "adolescen*"[All Fields] OR "infant*"[All Fields] OR "infant"[MeSH Terms] OR "infant, newborn"[MeSH Terms] OR "newborn*"[All Fields]

AND

"tuberculosi"[All Fields] OR "tuberculosis"[MeSH Terms] OR "tuberculosis"[All Fields] OR "tuberculoses"[All Fields] OR "tuberculosis s"[All Fields] OR "tuberculosis"[MeSH Terms] OR "TB"[All Fields] OR "Latent TB"[All Fields] OR "latent tuberculosis"[MeSH Terms] OR "Koch's Disease"[All Fields] OR "Mycobacterium infections"[MeSH Terms] OR "*Mycobacterium tuberculosis*"[MeSH Terms] OR "*Mycobacterium tuberculosis*"[All Fields]

AND

"risk factor*"[All Fields] OR "risk factors"[MeSH Terms] OR "socioeconomic factor*"[All Fields] OR "social determinants of health"[MeSH Terms] OR "social determinant*"[All Fields] OR "risk determinant*"[All Fields] OR "risk predictor*"[All Fields] OR "epidemiologic factor*"[All Fields] OR "epidemiologic factors"[MeSH Terms] OR "risk assessment*"[All Fields]

AND

"angola"[MeSH Terms] OR "angola"[All Fields] OR "angola s"[All Fields] OR ("bangladesh"[MeSH Terms] OR "bangladesh"[All Fields] OR "bangladesh s"[All Fields]) OR ("brazil"[MeSH Terms] OR "brazil"[All Fields] OR "brazil s"[All Fields] OR "brazils"[All Fields]) OR ("china"[MeSH Terms] OR "china"[All Fields] OR "china s"[All Fields] OR "chinas"[All Fields]) OR ("democratic people s republic of korea"[MeSH Terms] OR ("democratic"[All Fields] AND "people s"[All Fields] AND "republic"[All Fields] AND "korea"[All Fields]) OR "democratic people s republic of korea"[All Fields]) OR (("democrat"[All Fields] OR "democratic"[All Fields] OR "democratically"[All Fields] OR "democratization"[All Fields] OR "democratize"[All Fields] OR "democratized"[All Fields] OR "democratizing"[All Fields] OR "democrats"[All Fields]) AND ("republic"[All Fields] OR "republic s"[All Fields] OR "republics"[All Fields]) AND ("congo"[MeSH Terms] OR "congo"[All Fields])) OR ("ethiopia"[MeSH Terms] OR "ethiopia"[All Fields] OR "ethiopia s"[All Fields]) OR ("india"[MeSH Terms] OR "india"[All Fields] OR "india s"[All Fields] OR "indias"[All Fields]) OR ("indonesia"[MeSH Terms] OR "indonesia"[All Fields] OR "indonesia s"[All Fields] OR "indonesias"[All Fields]) OR ("kenya"[MeSH Terms] OR "kenya"[All Fields] OR "kenya s"[All Fields]) OR ("mozambique"[MeSH Terms] OR "mozambique"[All Fields] OR "mozambique s"[All Fields]) OR ("myanmar"[MeSH Terms] OR "myanmar"[All Fields] OR "myanmar s"[All Fields] OR "myanmars"[All Fields]) OR ("nigeria"[MeSH Terms] OR "nigeria"[All Fields] OR "nigeria s"[All Fields]) OR ("pakistan"[MeSH Terms] OR "pakistan"[All Fields] OR "pakistan s"[All Fields]) OR ("philippine"[All Fields] OR "philippines"[MeSH Terms] OR "philippines"[All Fields]) OR ("south africa"[MeSH Terms] OR ("south"[All Fields] AND "africa"[All Fields]) OR "south africa"[All Fields]) OR ("thailand"[MeSH Terms] OR "thailand"[All Fields] OR "thailand s"[All Fields]) OR ("uganda"[MeSH Terms] OR "uganda"[All Fields] OR "uganda s"[All Fields]) OR ("tanzania"[MeSH Terms] OR "tanzania"[All Fields] OR ("united"[All Fields] AND "republic"[All Fields] AND "tanzania"[All Fields]) OR "united republic of tanzania"[All Fields]) OR ("vietnam"[MeSH Terms] OR "vietnam"[All Fields] OR "vietnam s"[All Fields]) OR ("central african republic"[MeSH Terms] OR ("central"[All Fields] AND "african"[All Fields] AND "republic"[All Fields]) OR "central african republic"[All Fields]) OR ("congo"[MeSH Terms] OR "congo"[All Fields]) OR ("gabon"[MeSH Terms] OR "gabon"[All Fields]) OR ("lesotho"[MeSH Terms] OR "lesotho"[All Fields]) OR ("liberia"[MeSH Terms] OR "liberia"[All Fields] OR "liberia s"[All Fields]) OR ("mongolia"[MeSH Terms] OR "mongolia"[All Fields] OR "mongolia s"[All Fields]) OR ("namibia"[MeSH Terms] OR "namibia"[All Fields] OR "namibia s"[All Fields]) OR ("papua new guinea"[MeSH Terms] OR ("papua"[All Fields] AND "new"[All Fields] AND "guinea"[All Fields]) OR "papua new guinea"[All Fields]) OR ("sierra leone"[MeSH Terms] OR ("sierra"[All Fields] AND "leone"[All Fields]) OR "sierra leone"[All Fields]) OR ("zambia"[MeSH Terms] OR "zambia"[All Fields] OR "zambia s"[All Fields])

**Embase**

### Records obtained on June 10^th^ 2022- 2,602

(pediatric OR 'paediatric'/exp OR 'child'/exp OR 'child' OR 'children' OR 'adolescent'/exp OR 'infant'/exp OR 'infant' OR newborn) AND ('tuberculosis'/exp OR *'Mycobacterium tuberculosis* infection' OR 'tb (tuberculosis)' OR 'tb disease' OR 'tb infection' OR 'active tb' OR 'active tuberculosis' OR 'chronic tuberculosis' OR 'tuberculosis' OR 'tuberculous infection' OR 'latent tuberculosis'/exp OR '*Mycobacterium tuberculosis*'/exp OR 'bacillus tuberculosis' OR 'bacterium tuberculosis' OR 'koch bacillus' OR 'koch`s bacillus' OR 'mycobacteria tuberculosis' OR 'mycobacterium tuberculosin' OR *'Mycobacterium tuberculosis*' OR '*Mycobacterium tuberculosis* cultivation' OR *'Mycobacterium* tuberculosis *hominis'* OR *'Mycobacterium tuberculosis* isolation' OR 'mycobacterium tuberculosum' OR 'human tubercle bacillus' OR 'tubercle bacilli' OR 'tubercle bacillus' OR 'tuberculosis hominis, mycobacterium' OR 'tuberculosis, mycobacterium') AND ('democratic people republic of korea' OR 'angola'/exp OR 'angola' OR 'bangladesh'/exp OR 'bangladesh' OR 'democratic republic of congo ethiopia' OR 'brazil'/exp OR 'brazil' OR china OR india OR 'indonesia'/exp OR 'kenya'/exp OR 'mozambique'/exp OR 'mozambique' OR 'mocambique' OR 'myanmar'/exp OR 'nigeria'/exp OR 'pakistan'/exp OR 'philippines'/exp OR 'south africa'/exp OR 'thailand'/exp OR 'uganda'/exp OR 'tanzania'/exp OR 'tanzania' OR 'tanzania, united republic of' OR 'united republic of tanzania' OR 'viet nam'/exp OR 'central african republic'/exp OR 'centrafrican republic' OR 'central african empire' OR 'central african republic' OR congo OR 'gabon'/exp OR 'lesotho'/exp OR 'liberia'/exp OR 'mongolia'/exp OR 'namibia'/exp OR 'namibia' OR 'papua new guinea'/exp OR 'new guinea' OR 'papua' OR 'papua new guinea' OR 'papua and new guinea' OR 'zambia'/exp OR 'zambia') AND ('risk factors' OR 'socioeconomics'/exp OR 'economic value of life' OR 'health care, indigent' OR 'indigent health care' OR 'medical indigency' OR 'social economic aspect' OR 'social economics' OR 'social-economic factor' OR 'socio-economic aspect' OR 'socio-economic factor' OR 'socio-economics' OR 'socioeconomic aspect' OR 'socioeconomic factor' OR 'socioeconomic factors' OR 'value of life' OR 'social determinants of health' OR 'social determinant' OR 'risk determinant' OR 'epidemiologic factors' OR 'risk assessment')

Filters applied- #1 AND (1990:py OR 1991:py OR 1992:py OR 1993:py OR 1994:py OR 1995:py OR 1996:py OR 1997:py OR 1998:py OR 1999:py OR 2000:py OR 2001:py OR 2002:py OR 2003:py OR 2004:py OR 2005:py OR 2006:py OR 2007:py OR 2008:py OR 2009:py OR 2010:py OR 2011:py OR 2012:py OR 2013:py OR 2014:py OR 2015:py OR 2016:py OR 2017:py OR 2018:py OR 2019:py OR 2020:py OR 2021:py OR 2022:py) AND ('article'/it OR 'article in press'/it OR 'conference paper'/it OR 'preprint'/it OR 'short survey'/it) AND ('case control study'/de OR 'case report'/de OR 'case study'/de OR 'clinical article'/de OR 'cohort analysis'/de OR 'comparative study'/de OR 'controlled clinical trial'/de OR 'controlled study'/de OR 'cross sectional study'/de OR 'human'/de OR 'major clinical study'/de OR 'multicenter study'/de OR 'normal human'/de OR 'observational study'/de OR 'outcomes research'/de OR 'pilot study'/de OR 'prospective study'/de OR 'quantitative study'/de OR 'questionnaire'/de OR 'randomized controlled trial'/de OR 'randomized controlled trial topic'/de OR 'retrospective study'/de)

Supplement Table I Quality assessment results for cohort studies in the systematic review.

| Author | Selection | | | | Comparability | | Outcome | | | Total score |
| --- | --- | --- | --- | --- | --- | --- | --- | --- | --- | --- |
|  | Representativeness of the exposed cohort | Selection of the non-exposed cohort | Ascertainment of exposure | Demonstration that outcome of interest was not present at the start of study | Comparability: age and sex | Comparability: additional factors | Ascertainment of outcome | Follow-up long enough | Adequacy of follow-up |  |
| E. W. Bunyasi, 2019 | * | * | * | * | * | * | * | * | * | 9 |
| Daniel Mumpe-Mwanja,  2015 | * | - | * | * | * | - | * | - | - | **5** |

Supplement Table II Quality assessment results for case-control studies in the systematic review.

| **Author** | **Selection** | | | | **Comparability** | | **Exposure** | | | **Total score** |
| --- | --- | --- | --- | --- | --- | --- | --- | --- | --- | --- |
|  | Is the case definition adequate? | Representativeness of the cases | Selection of Controls | Definition of Controls | Comparability:  Age and Gender | Comparability:  Other factors | Ascertainment of exposure | Same method of ascertainment for cases and controls | Non-Response rate |  |
| Nkosana Jafta, 2019 | * | * | * | * | * | - | * | * | - | **7** |
| J. Jubulis,2014 | * | * | * | - | * | * | * | * | - | **7** |
| Hilary Stevens, 2014 | * | * | * | * | * | - | * | * | - | **7** |
| Karim MR, 2012 | * | * | - | * | * | * | * | * | - | **7** |
| Somdatta Patra, 2012 | * | * | - | * | * | - | * | * | - | 6 |
| Karim MR, 2012 | * | * | - | * | * | - | * | * | - | 6 |
| V.K. Chadha, 2011 | * | * | * | * | * | - | * | * | - | **7** |

Fig VI Forest plot, Galbraith plot and Funnel plot for gender as risk factor

Fig VII Forest plot, Galbraith plot and Funnel plot for presence of windows as risk factor

Fig VIII Forest plot and Galbraith plot for mother’s education as risk factor

Fig IX Forest plot, Galbraith plot and Funnel plot for no. of rooms as risk factor

Fig X Forest plot, Galbraith plot and Funnel plot for child’s education as risk factor

Fig XI Forest plot, Galbraith plot and Funnel plot for BCG vaccination as risk factor

Fig XII Forest plot, Galbraith plot and Funnel plot for older age as risk factor
